# RETRACTED: D’Agostino et al. Molecular Mechanisms at the Basis of Pharmaceutical Grade *Triticum vulgare* Extract Efficacy in Prompting Keratinocytes Healing. *Molecules* 2020, *25*, 431

**DOI:** 10.3390/molecules31183311

**Published:** 2026-09-18

**Authors:** Antonella D’Agostino, Anna Virginia Adriana Pirozzi, Rosario Finamore, Fabrizia Grieco, Massimiliano Minale, Chiara Schiraldi

**Affiliations:** 1 Department of Experimental Medicine, Section of Biotechnology, Medical Histology and Molecular Biology, University of Campania “Luigi Vanvitelli”, 80138 Naples, Italy; antonella.dagostino@unicampania.it (A.D.); annavirginiaadriana.pirozzi@unicampania.it (A.V.A.P.); rosario.finamore@unicampania.it (R.F.); 2 Farmaceutici Damor, 80145 Naples, Italy; fabri.gri1224@hotmail.it (F.G.); massimiliano.minale@farmadamor.it (M.M.)

The Journal retracts the article “Molecular Mechanisms at the Basis of Pharmaceutical Grade *Triticum vulgare* Extract Efficacy in Prompting Keratinocytes Healing” [1], cited above.

Following publication, concerns were brought to the attention of the Editorial Office regarding the integrity of a figure presented in this article [1].

In accordance with the Journal’s standard procedures, the Editorial Office and Editorial Board conducted an investigation which identified indications of inappropriate image editing and duplication within Figure 5 of this publication [1]. Although the authors cooperated with the investigation, the Editorial Board concluded that the explanations and the supporting materials provided could not satisfactorily resolve the identified concerns.

As a consequence, the Editorial Board decided to retract this publication in accordance with MDPI’s retraction policy.

This retraction was approved by the Editor-in-Chief of the journal *Molecules*.

The authors have been informed of this retraction but did not provide a comment on this decision.
